# A binary method for simple and accurate two-dimensional cursor control from EEG with minimal subject training

**DOI:** 10.1186/1743-0003-6-14

**Published:** 2009-05-06

**Authors:** Turan A Kayagil, Ou Bai, Craig S Henriquez, Peter Lin, Stephen J Furlani, Sherry Vorbach, Mark Hallett

**Affiliations:** 1 National Institute of Neurological Disorders and Stroke, Bethesda, MD 20892, USA; 2 Duke University Department of Biomedical Engineering, Durham, NC 27708, USA; 3 Georgetown University School of Medicine, Washington, DC 20057, USA; 4 Virginia Commonwealth University Department of Biomedical Engineering, Richmond, VA 23284, USA

## Abstract

**Background:**

Brain-computer interfaces (BCI) use electroencephalography (EEG) to interpret user intention and control an output device accordingly. We describe a novel BCI method to use a signal from five EEG channels (comprising one primary channel with four additional channels used to calculate its Laplacian derivation) to provide two-dimensional (2-D) control of a cursor on a computer screen, with simple threshold-based binary classification of band power readings taken over pre-defined time windows during subject hand movement.

**Methods:**

We tested the paradigm with four healthy subjects, none of whom had prior BCI experience. Each subject played a game wherein he or she attempted to move a cursor to a target within a grid while avoiding a trap. We also present supplementary results including one healthy subject using motor imagery, one primary lateral sclerosis (PLS) patient, and one healthy subject using a single EEG channel without Laplacian derivation.

**Results:**

For the four healthy subjects using real hand movement, the system provided accurate cursor control with little or no required user training. The average accuracy of the cursor movement was 86.1% (SD 9.8%), which is significantly better than chance (p = 0.0015). The best subject achieved a control accuracy of 96%, with only one incorrect bit classification out of 47. The supplementary results showed that control can be achieved under the respective experimental conditions, but with reduced accuracy.

**Conclusion:**

The binary method provides naïve subjects with real-time control of a cursor in 2-D using dichotomous classification of synchronous EEG band power readings from a small number of channels during hand movement. The primary strengths of our method are simplicity of hardware and software, and high accuracy when used by untrained subjects.

## Background

Interfaces which interpret user brain activity to effect some output have potential applications to many fields, including aiding individuals with disabilities to control devices and communicate. There are several different approaches to creating brain-computer interfaces (BCIs). The most invasive method involves single-unit recording, where arrays of implanted electrodes are used to record trains of action potentials from individual neurons. Single-unit recordings have been used successfully to provide fairly sophisticated control [1]. Implantation of the electrodes, however, requires surgery, and a practical clinical implementation of single-unit recordings will require methods that can telemeter the data without transcutaneous wires [2]. Electrocorticography (ECoG) is less invasive than single-unit recording as it uses electrodes placed directly on the cortical surface, but at a cost of lower spatial resolution. The least invasive method of brain-computer interface uses electroencephalography (EEG) recording where external electrodes are placed on the scalp. EEG signals have even lower spatial resolution than ECoG and typically have lower signal-to-noise ratios than other BCI methods. Control methods used in EEG and ECoG BCIs are generally similar (see, for example, [3]), and are distinct from those used in single-unit recording BCIs. Other BCI techniques such as with magnetoencephalography (MEG) and functional magnetic resonance imaging (fMRI) do not seem practical.

One common method of EEG control relies on power changes. Event-related desynchronization (ERD) is a reduction in EEG signal power within a certain frequency band as a result of a particular event. For example, when a subject is making a hand movement, a reduction in sensorimotor rhythm power might be observed in the subject's contralateral sensorimotor cortex. Desynchronization can be used to control a computer cursor in one dimension (1-D); often the subject will try to control his or her rhythm to move a cursor in one dimension on a screen [4-7]. In some paradigms, while the subject controls the cursor movement in one dimension, the cursor travels in the other dimension at a constant rate towards a group of targets on one side of the screen [6,7]. Guiding the cursor in this way allows one of the targets to be selected when the cursor reaches the edge of the screen. Fewer targets provide fewer choices, while more targets decrease accuracy [6]. Chains of selections using 1-D control may be strung together sequentially to facilitate selection of one choice from a large group of choices, with each level of selection further narrowing the field of remaining choices until the final choice is made. This technique is called a decision tree. One possible application of a decision tree is virtual keyboard control [8].

Another method of EEG control, which has also been applied to virtual keyboards [9-12], uses evoked potential detection to allow the user to select one target of several. In a P-300 evoked potential paradigm, target choices are typically presented in a group and then are highlighted (individually or in smaller groups) until the computer can determine which target, when highlighted, elicits a P-300 evoked potential. The P-300 is a positive wave that occurs about 300 ms after the presentation of a meaningful stimulus. As such, it is taken as a sign of the subject's recognition of the stimulus as being particularly relevant. The computer then concludes that this target most likely represents the choice that the subject wishes to make. A steady state visual evoked potential (SSVEP) paradigm relies on targets which flicker at different rates, thereby triggering SSVEPs at different frequencies. The computer detects the SSVEP frequency to determine which target is salient.

Several different approaches have been taken to provide two-dimensional (2-D) cursor control from EEG. Wolpaw et al. measured band power from 64 channels, from both hemispheres and two different bands simultaneously, with each band controlling a different dimension of the cursor movement, and with the two hemispheres making opposite-signed contributions to the movement [13]. An earlier study by Wolpaw et al. used the sum and difference of band power measurements from two channels of bipolar EEG from the two hemispheres to provide vertical and horizontal cursor control, respectively [14]. Evoked potential methods have also been employed, including the four-channel P-300 detection method of Piccione et al. [15], and the 12-channel SSVEP method of Trejo et al. [7]. In the P-300 paradigm, the user chooses the direction of cursor movement by attending to one of four direction arrows which are sequentially highlighted before each move. In the SSVEP paradigm, the user chooses the direction of cursor movement by attending to one of four flickering stimuli, each of which flickers at a different frequency. Geng et al. [16] describe a "parallel" BCI system under which two bits of information may be obtained simultaneously from EEG during real or imagined hand and foot movement. Although they did not apply this system to real-time 2-D cursor control, it is easy to envision such an application.

Most EEG-based BCIs use multiple channels of EEG recording. Because of the time required for electrode setup and the associated hardware to process multiple channels, there is an advantage to reduce the number of channels. In this paper, we investigate the ability to achieve real-time 2-D cursor control using a single channel of EEG with four additional channels to allow Laplacian derivation. The results from six subjects show that 2-D control can be achieved with good accuracy and relatively low computational demand using an optimally placed single electrode with Laplacian derivation, despite minimal subject training. The ability to achieve good control rapidly with a single electrode using Laplacian derivation may provide another practical option in the continuing development of EEG-based BCI assistive technologies. The aim of this study was to identify a method for reliable EEG-based BCI control that can be implemented with minimal subject training and relative simplicity of hardware and software.

## Methods

### Paradigm design

To provide robust single-channel control, we implemented a synchronous binary approach to 2-D cursor control. Synchronous control uses a pre-defined time window for each user response so that the computer does not need to determine when a user response occurs, but only into which class each user response falls. Binary control refers to a situation under which each response must be classified into one of only two classes, as contrasted with control where a response can be classified into one of a greater number of classes or ignored altogether. Synchronous binary classification is the simplest possible classification using EEG, and we hypothesized that this simplicity would yield high cursor movement accuracy.

The binary approach works as follows. The cursor moves in discrete steps, and each step is in one of four directions (up, down, left, right) as selected by the user through his or her EEG signal. To select a direction, the user effectively answers "yes" or "no" two times in a row, performing continuous right-hand movement to answer "yes," or abstaining from such movement to answer "no." The user has a short time to give each answer, during which the resultant ERD causes a power change in the EEG signal. The computer program measures the EEG power from a single optimum channel and frequency band over the pre-defined time window of the subject's answer. If the power is above a certain threshold the software algorithm interprets the answer as a "no," and if the power is below the threshold the software algorithm interprets the answer as a "yes." The program determines the threshold value prior to the user's first game by presenting a series of "yes" or "no" prompts that the user obeys directly, and using the associated power measurements from the appropriate location/band to optimize classification accuracy. This threshold determination does not have to be repeated before each game.

**Figure 1:**
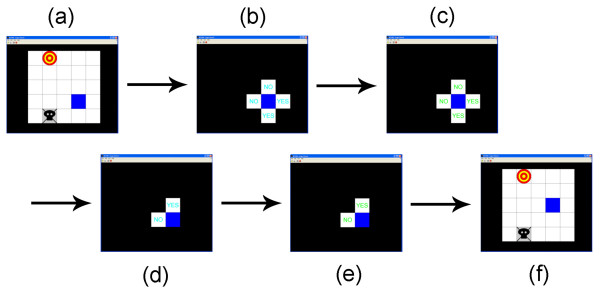
**Sequential screen shots of the 2-D cursor control paradigm**. (a) A game grid is displayed showing a cursor, target, and trap. (b) All squares except those adjacent the cursor are masked, and cyan prompts are displayed in the adjacent squares. The subject begins a continuous right hand movement. (c) After brief pause, the prompts turn green to indicate the period during which the subject should respond. The user responds "yes" by continuing the right hand movement, or "no" by ceasing the movement. In the example shown here, the user gives a "no" response. (d) The user's response narrows the choices of directions from four to two, and the prompting process is repeated starting with cyan prompts. (e) The cyan prompts are again followed by green prompts during which the subject responds. In this example, the user responds "yes." (f) Finally, the subject's response uniquely determines the cursor movement direction, and the mask is lifted while the cursor slides in the chosen direction. The entire process (a)-(f) then repeats for the next cursor move, and so on until the target is obtained, the trap is hit, or too many moves have been made. The exact timing of each step is set to make the particular subject comfortable, but a typical duration for one complete cursor move is about 15 s.

Under the 2-D cursor control paradigm, a cursor moves among squares of a grid towards a target while avoiding a trap. Sequential screen shots of one cursor move are shown in Figure 1. The subject is presented with the game grid, and is allowed to blink, shift gaze, and strategize for the next move. After presentation, everything but the cursor and four adjacent squares are blacked out, and a prompt is presented in each of the possible movement directions. For all but one of the studies presented here, EEG signals were recorded with the subject making hand movements. One example is presented in which control was performed with only motor imagery. When movements are used, the subject initiates control by making continuous right hand movement. The prompts remain cyan for a short time to allow the subject to interpret the prompt in the desired movement direction, and then the prompts turn green. While the prompts are green, the subject executes the desired task. To select a direction showing a "yes" prompt, the subject continues the right hand movement. To select a direction showing a "no" prompt, the subject ceases the movement and remains motionless throughout the green prompt. In either case, the subject must fixate on the prompt, remain relaxed, and not blink to avoid artifacts while the prompt is green. Once the program determines the first response (first bit), it eliminates the two rejected directions, and repeats the prompting process. After the second response (second bit), the game grid again becomes visible, and the cursor moves to the new position. The entire process for one (two-bit) cursor move takes about 15 s. When the game is played without hand movements (as in one of our supplementary tests), the subject is asked instead to imagine a movement. When playing the game using motor imagery, the threshold-setting and control tasks are performed as normal.

Additional file 1: ExampleVideo is a short video clip of the 2-D cursor control game. This file is provided only to demonstrate the appearance of the game.

While on any given movement the cursor moves in only one direction, the control is two-dimensional rather than one-dimensional because the direction of each movement can be any one of four choices in two dimensions. This is analogous to the two-dimensional control achieved by the P-300 detection method of Piccione et al. [15], which also uses a series of single cursor movements, each in one of four directions. Whereas Piccione's method relies on sequential emphasis of four stimuli to obtain the two bits of information required for each cursor move, our method obtains the first and second bits sequentially through two user selections, which together uniquely identify both the dimension and direction of each cursor move. This two-dimensional control is distinct from one-dimensional control, wherein the computer restricts the dimension of cursor movement, and the user is free to control only the direction of the movement.

The cursor control game incorporates several additional features. These include automatic recordkeeping, game scoring to hold player interest, and an optional adaptive threshold feature (which was used only for Subject F, as discussed below). Furthermore, the program avoids superfluous prompts; if the cursor is at an edge of the grid and the first prompt can uniquely determine cursor movement direction, then only one prompt is provided.

### Study procedures

A Neuroscan Synamp 1 amplifier (Neuroscan Inc., El Paso, TX, USA) amplified the EEG signal from 29 electrodes. The 29 electrodes sampled at 250 Hz from FP1, F3, F7, C3A, C1, C3, C5, T3, C3P, P3, T5, O1, FP2, F4, F8, C4A, C2, C4, C6, T4, C4P, P4, T6, O2, FZ, CZA, CZ, PZA, and PZ in an elastic cap (Electro-Cap International, Inc., Eaton, OH, USA). The recordings from a maximum of five of these 29 electrodes were used for each subject's cursor control, although all 29 electrodes were used once per subject for the initial channel/bin optimization step, which did not need to be repeated thereafter. A Hewlett-Packard workstation converted the amplified analog signal to a digital signal.

We determined the optimum single electrode location and frequency band for control for each subject from offline analysis of EEG recordings. First, each subject performed the threshold-setting task (although no threshold was set at this point) wherein single predetermined yes/no prompts were presented sequentially. This threshold-setting task consisted of 30 prompts, composed of 15 "yes" and 15 "no" prompts randomly interspersed. An offline feature analysis of the resultant EEG recordings was performed to identify the location and band for which power measurements provided the greatest yes/no class separability. Once the optimum location and band were identified, these were used for all subsequent testing with the subject. Thus, this optimization step, which required a relatively large number of electrodes (all 29 were analyzed), only needed to be performed once per subject, and then a reduced number of electrodes could be used (five electrodes if using Laplacian derivation, or one electrode if not).

Once the optimum location and band were identified, each subject repeated the threshold-setting task, and the power in the optimum location/band was again computed (now using the reduced number of electrodes). These measurements were used to set an optimum threshold. For these experiments, the threshold-setting task again consisted of 30 prompts, composed of 15 "yes" and 15 "no" prompts randomly interspersed. Completion of the entire threshold-setting task took less than 5 minutes. The threshold determined from this task was used for the subsequent 2-D cursor control task. Each subject repeated the threshold-setting task multiple times to practice his or her control strategy. However, each time the task was repeated, the program discarded all previously obtained data. Thus, the threshold set by the program was based solely on the 30 prompts from the subject's most recent performance of the threshold-setting task.

Finally, each subject performed the 2-D cursor control task. The program interpreted intended cursor movement direction online in real-time by comparing measured powers to the optimum threshold. The program also tagged the EEG recordings with the interpreted yes/no answers. An electromyography (EMG) channel recorded right hand movement during the cursor control task. The EMG signal was sampled at 250 Hz from a bipolar surface electrode located over each subject's right wrist extensor muscles. Visual inspection of the EMG recording was used to quantify the control accuracy through post-hoc offline analysis.

### Computational method

For all prompts in the threshold-setting and cursor control tasks, the time over which the subject gave each yes or no answer had duration 2 s. Band power measurements were computed for the final 1.5 s of this time window only, to allow for subject response time. Power was determined using the Welch estimation method with FFT length (nonequispaced fast Fourier transform) of 64 and a Hamming window with 50% overlap [17]. The sampling rate of this study was 250 Hz, and the frequency resolution was about 4 Hz. For all measurements, the EEG signal was referenced using Laplacian derivation to reduce error. This means that the EEG signal was referenced from each electrode to the average of the potentials from the nearest four orthogonal electrodes. For example, the program referenced the C3 channel to the average of C1, C3A, C5, and C3P, each of which was about 3 cm from C3, and calculated band power on C3 for the referenced signal.

To determine the optimum spatial location and frequency band for discrimination, we conducted a feature analysis by calculating Bhattacharyya distances from power measurements. Frequency bands were 4 Hz wide, corresponding to the 4 Hz resolution of the power measurement. We measured power using the Welch method for each yes/no response, for each EEG channel. Then, for each channel/bin pair, we calculated a Bhattacharyya distance based on the power measurements for all of the responses from both the "yes" and "no" classes. Higher Bhattacharyya distances corresponded to better yes/no class separability, and identified the more effective channels and frequency bands for control. We calculated each Bhattacharyya distance according to (1), where *M*_*i *_and Σ_*i *_are the mean vector and covariance matrix of class *i *( = 1,2), respectively [18]. As we measured the Bhattacharyya distance for each channel and frequency bin, *M*_*i *_is a scalar.

(1)

After we identified the optimum location and frequency band (only done once per subject), we used these in our threshold-setting program, which no longer needed all EEG channels. This program measured power in the optimum location/band while the subject performed the threshold-setting task. After the task was complete, a receiver operating characteristics (ROC) curve was generated by determining the true positive and false positive fractions that would result from various values of threshold. Here, "true positive fraction" refers to the fraction of intended "yes" answers that the program would interpret as "yes" answers given the particular threshold value (this is equivalent to sensitivity). "False positive fraction" is the fraction of intended "no" answers that the program would interpret as "yes" answers (this is equivalent to 1 – specificity). The threshold-setting program chose the optimal threshold as that which minimized the distance defined in (2).

(2)

Additional file 2: Overview summarizes the most important steps of the binary control computational method. The file shows examples of recorded EEG signals, and indicates how these signals can be classified based on their power spectral densities into "yes" and "no" classes. The file demonstrates the correspondence between higher Bhattacharyya distances and better class separability, and shows how choosing the optimum location/band can yield a high-quality ROC curve, from which a threshold can be set and subsequently used to achieve good control in the 2-D cursor control task.

To quantitatively assess the accuracy of the cursor control, we analyzed the recordings from the control task offline following each subject's session. We compared our program's yes/no interpretations with the recorded right wrist EMG trace to explicitly determine whether each classification and cursor move was correct.

For motor imagery, no EMG signal was available for comparison, so we assessed the accuracy of yes/no classification from one of the threshold-setting task recordings. We divided the prompts into a training set consisting of the first 7 "yes" and first 7 "no" responses, and a testing set consisting of the last 8 "yes" and last 8 "no" responses. We used the training set to calculate an optimum threshold, which we then applied to the testing set to classify its responses. Because we knew the correct classifications of the responses, we were able to quantify the classification accuracy. We also used the entire threshold-setting task to set an optimum threshold with which the subject played the cursor control game. We then asked the subject to qualitatively evaluate her control after playing the game.

### Subjects and data acquisition

We tested the paradigm with four healthy subjects using hand movement. Subjects included three females and one male, with ages ranging from 24–55 years. Subject A was female, age 53 years. Subject B was female, age 55 years. Subject C was female, age 24 years. Subject D was male, age 32 years.

We also carried out several supplementary tests. Subject B performed our paradigm using motor imagery. This followed Subject B's session using real movement. Subject E, a primary lateral sclerosis (PLS) patient, performed our paradigm using hand movement. PLS is a motor neuron disease, the symptoms of which include slowly progressive spasticity of unknown cause without clinical signs of lower motor neuron loss. Pathological studies show degeneration of the corticospinal tracts. Subject E was female, age 58 years, with the disease for 11 years. She was identified as a PLS-A patient with loss of motor-evoked potentials by transcranial magnetic stimulation, and her right finger tapping rate was 3.6 taps/s, which was significantly lower than healthy controls of 5.8 taps/s [19]. Subject F performed our paradigm using hand movement, but with no Laplacian derivation referencing of the EEG channels. Subject F was male, age 23 years. We also performed a post-hoc offline analysis of data from Subject A with the Laplacian derivation removed.

None of the subjects had previous BCI experience. All subjects were right-handed according to the Edinburgh inventory [20]. All subjects gave written informed consent for the protocol, which was approved by the institutional review board.

We accomplished the real-time EEG data acquisition and processing using a Matlab-based self-developed hardware and software system. The self-developed Matlab scripts accessed the digital signal and performed the power spectral estimation. Finally, the scripts decoded the power spectral signal to drive the cursor movement.

## Results

### Feature analysis

**Figure 2:**
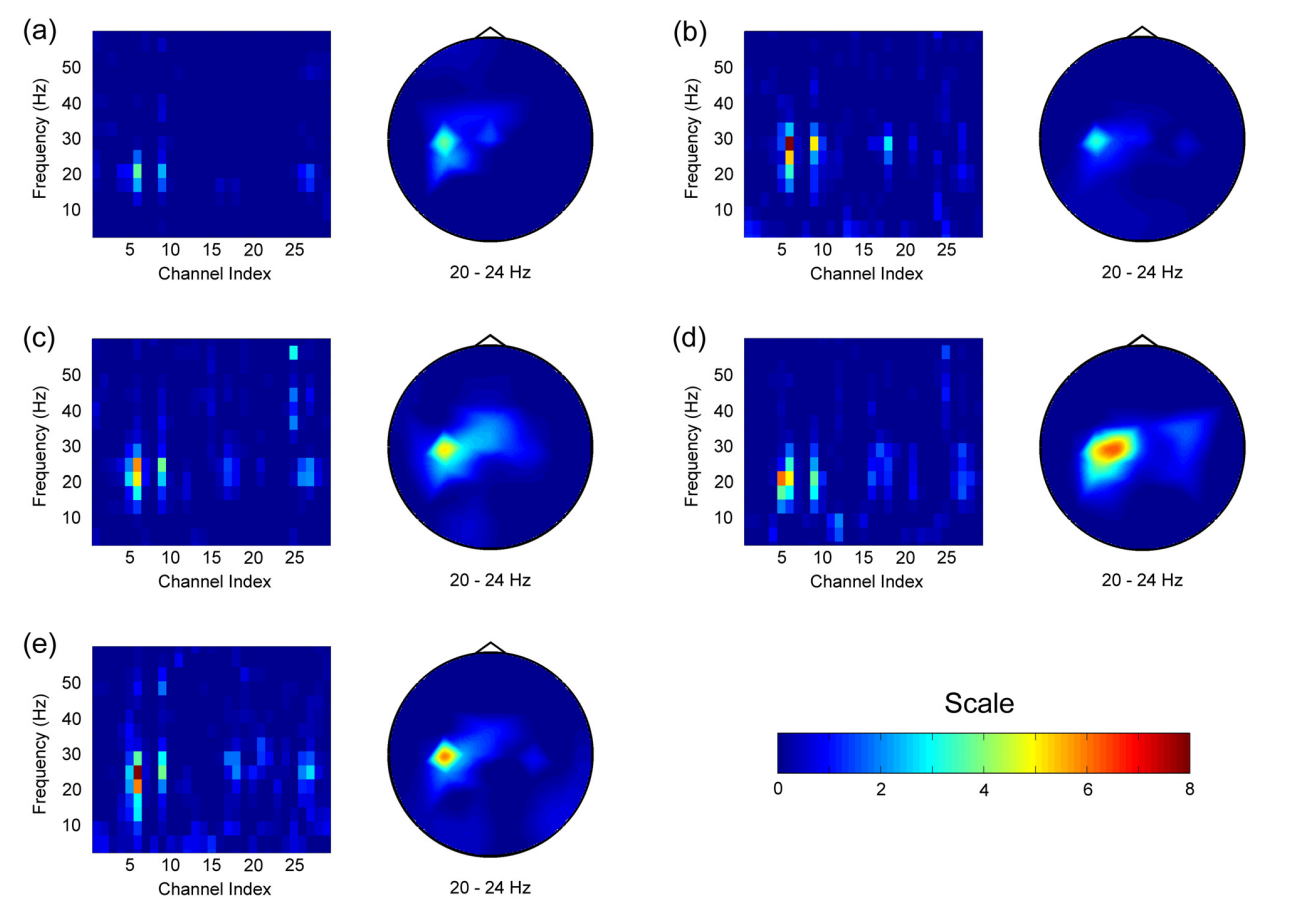
**Bhattacharyya distance plots for real movement**. Higher values indicate greater class separability. (a) Subject A – healthy subject. Left: Channel-frequency plot, showing that the best EEG power-based classification may be obtained from the channel 6, or C3, electrode, and the 20–24 Hz frequency bin. Right: Head topography plot for only the 20–24 Hz frequency bin, showing that the most relevant signal is localized over the left sensorimotor cortex. This is the location of the C3 electrode. (b) Subject B – healthy subject. Left: Channel-frequency plot. Right: Head topography plot for the 20–24 Hz bin. (c) Subject C – healthy subject. Left: Channel-frequency plot. Right: Head topography plot for the 20–24 Hz bin. (d) Subject D – healthy subject. Left: Channel-frequency plot. Right: Head topography plot for the 20–24 Hz bin. (e) Subject E – PLS patient. Left: Channel-frequency plot. Right: Head topography plot for the 20–24 Hz bin.

Figure 2 shows channel-frequency and head topography plots of Bhattacharyya distances for Subjects A-E using hand movement. For all subjects, including the PLS patient (Subject E), the largest Bhattacharyya distances were localized over the left sensorimotor cortex, contralateral to the hand being moved, and were located in the beta frequency band, consistent with expectations about the sensorimotor rhythm. To attempt accurate control without the need for channel/bin calibration on an individual-by-individual basis, we chose the C3 electrode and 20–24 Hz frequency band as the optimum channel/bin for Subjects A, B, C, and E, since none of their Bhattacharyya plots differed extremely from this pattern.

For Subject D, we modified our threshold-setting program to automatically choose the best channel/bin as that which yielded the smallest minimum value of the distance defined by (2). In this way, we effectively automated the feature analysis by integrating it into the threshold-setting program, eliminating the need for the calculation of Bhattacharrya distances, but requiring that all 29 electrodes be used during the threshold-setting task. Our modified program chose the C1 electrode (channel 5) and the 20–24 Hz frequency bin for optimum control for Subject D. This selection is clearly consistent with the subject's Bhattacharyya plots.

### Binary 2-D cursor control with hand movement

**Figure 3:**
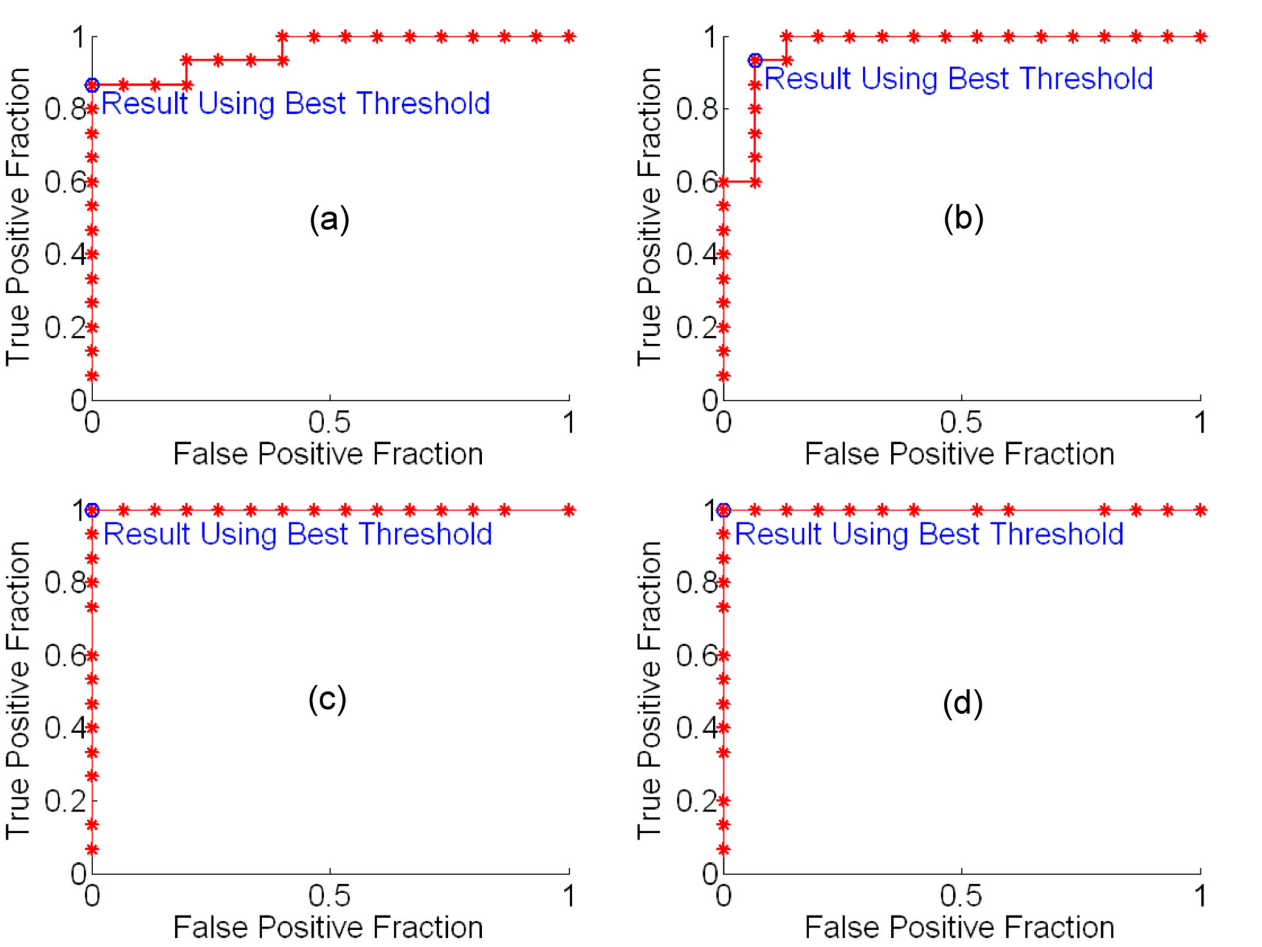
**ROC curves from the four healthy subjects using real movement**. For each subject, the curve shown was obtained from the threshold-setting task prior to the subject's first game. (a) Subject A. (b) Subject B. (c) Subject C. (d) Subject D. All curves demonstrate very good classification; for Subjects C and D classification is perfect.

For all four healthy subjects using hand movement, the threshold-setting task robustly classified the "yes" and "no" responses. Figure 3 shows the ROC curves generated by the threshold-setting task that immediately preceded each subject's first session of cursor control. For all curves, the optimum threshold clearly yielded a low value of the distance defined in (2).

After the threshold-setting task, each subject performed the 2-D cursor control task with hand movements. Subjects A, B, and D achieved good cursor control immediately. Subject C initially had more trouble with control, with an overall accuracy of 54.5% for her first 22 cursor moves (from 51.9% true positive and 92.3% true negative percentages for her first 40 yes/no answers). She then took a short break before proceeding. Following this break, her control accuracy improved. The results from the four subjects after they had adjusted to the cursor control task are summarized in Table 1. For all subjects except Subject C, these results are from the first attempt at the cursor control task following the threshold-setting task. For Subject C, the results are from the second attempt at the control task following the short break.

**Table 1:** 2-D cursor control results

Subject	Positives		Negatives		Moves		# Bits	# Moves	TP%	TN%	CM%
	True	False	True	False	Correct	Incorrect					
A	39	5	57	3	47	8	104	55	92.9%	91.9%	**85.5%**

B	44	12	33	4	43	16	93	59	91.7%	73.3%	**72.9%**

C	14	2	18	0	18	2	34	20	100.0%	90.0%	**90.0%**

D	29	1	17	0	24	1	47	25	100.0%	94.4%	**96.0%**

Results from the four healthy subjects (one session per subject) using real hand movement with Laplacian derivation. For all subjects except Subject C, results are from the first session of game play following the initial threshold-setting task. For Subject C, there was a short intervening session of practice game play (see text). Positives are subjects' answers that the program classified as yes answers; Negatives were classified as no answers. True classifications were correct, and False classifications were incorrect. Correct Moves are cursor moves for which movement was in the direction intended by the subject; Incorrect Moves were in an unintended direction. The total number of yes/no answers given during each subject's games is # Bits, the sum of True Positives, False Positives, True Negatives, and False Negatives. The total number of cursor moves during each subject's games is # Moves, the sum of Correct Moves and Incorrect Moves. TP% is the true positive percentage, the percentage of intended yes answers that the program correctly classified. TP% is given by True Positives/(True Positives + False Negatives). TN% is the true negative percentage, the percentage of intended no answers that the program correctly classified. TN% is given by True Negatives/(True Negatives + False Positives). Chance level is 50% for both TP% and TN%. The false negative and false positive percentages (not shown) may be calculated by subtracting TP% and TN% from 100%, respectively. The correct bit percentage (not shown) may be calculated as 100% × (True Negatives + True Positives)/# Bits. CM% is the percentage of all cursor moves that were in the correct direction. Chance level for CM% is 31.2% (greater than 25% because when the cursor is at a grid edge, sometimes only one yes/no answer is required for a cursor move).

Overall, the average of the subjects' cursor movement accuracies was 86.1%, with a standard deviation of 9.8%. This control was significantly greater than chance (p = 0.0015).

### Supplementary tests

For some of the subjects, additional test were performed to help determine the robustness of the single channel system when no or weak movements were used and when the Laplacian referencing was removed.

#### Subject B: 2-D control with motor imagery (no hand movement)

To gain a sense of whether our method would be effective for individuals who were unable to make hand movements, we performed a test of the paradigm for motor imagery with a single subject. We asked Subject B to repeat the threshold-setting and cursor control tasks using motor imagery immediately following her performance of the cursor control task using real movement.

**Figure 4:**
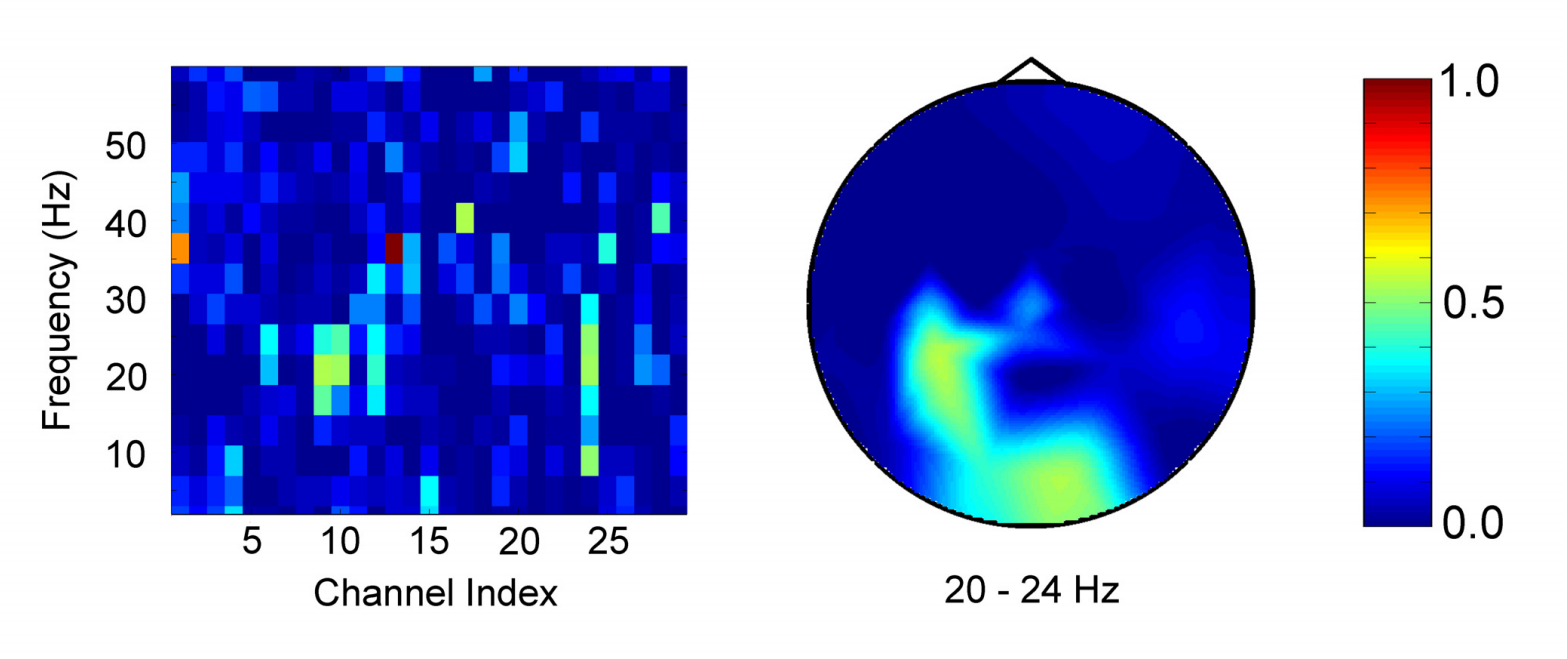
**Bhattacharyya plots for Subject B using motor imagery**. Left: Channel-frequency plot. Right: Head topography plot for the 20–24 Hz bin.

Figure 4 shows Bhattacharyya distance plots for Subject B using motor imagery. As expected, it was difficult to confidently determine an optimum channel/bin, so we used the same channel/bin as with real movement for the sake of parsimony.

**Figure 5:**
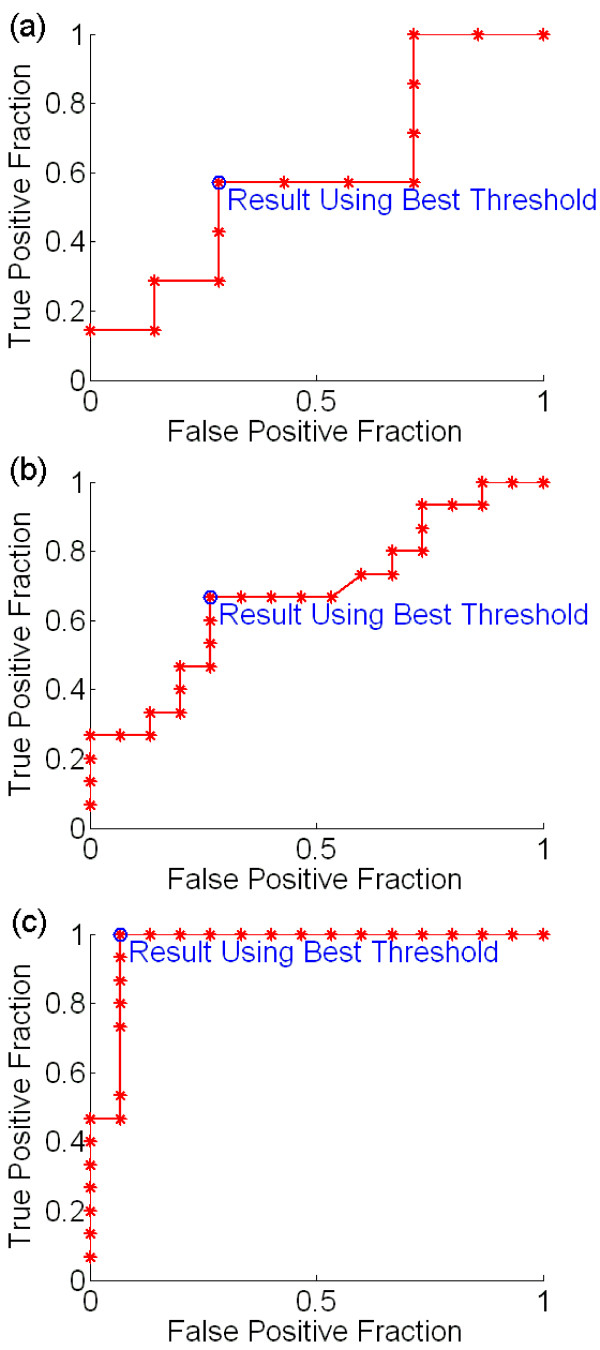
**Supplementary ROC curves**. (a) Subject B, using motor imagery, with curve based on training set only (see text). (b) Subject B, using motor imagery, with curve based on entire threshold-setting task. (c) Subject E, PLS patient, using real hand movement.

As described above, because no EMG signal was available with which to compare classification, accuracy with motor imagery was quantified using only the threshold-setting task divided into training and testing sets. Figure 5(a) shows the ROC curve from threshold optimization using the training set. Using this threshold, the classification accuracy for the testing set was as follows: 50.0% true positive percentage (chance = 50.0%), 87.5% true negative percentage (chance = 50.0%). Because there are no cursor moves in the threshold-setting task, no correct cursor move percentage could be calculated. However, this value may be estimated by assuming that intended yes and no answers are equally likely, and that all intended moves are equally likely. Under these assumptions, the average classification accuracy is the average of the true positive and true negative fractions (true negative fraction is the fraction of intended "no" answers correctly classified as "no", which is equivalent to specificity). The average number of bits per cursor move for the 5-by-5 grid is 1.68. The estimated correct cursor movement percentage *CM*% is then given by (3).

(3)

From (3), the estimated cursor movement accuracy for Subject B using motor imagery was 53.3% (chance = 31.2%).

Figure 5(b) shows the ROC curve from the threshold optimization based on the entire threshold-setting task. Using the threshold set from this ROC curve, the subject played the cursor control game. Although we could not quantify cursor movement accuracy, we asked the subject to qualitatively evaluate her control. She reported that she felt she had some control over the cursor movement, although she found the task frustrating.

#### Subject E: 2-D control with weak movement

We performed another supplementary test of the paradigm with a PLS patient, Subject E, using real movement. Subject E was able to make hand movements, but because of her disease, her movement was very weak.

The ROC curve for Subject E is shown in Figure 5(c). The curve again indicates robust threshold setting, with a very small value for the distance defined in (2). We analyzed the recordings from Subject E's performance of the cursor control task in the same way as the recordings from the healthy subjects with real movement. However, because Subject E's movements were very weak, the EMG signal (used as the reference for intended user responses) was often difficult to interpret visually. Thus, the results reported here for Subject E are approximate.

Subject E made 85 cursor moves, consisting of 60 correct moves and 25 incorrect moves. The total number of yes/no answers comprising these moves was 118, consisting of 57 true positives, 14 false positives, 36 true negatives, and 11 false negatives. This corresponds to an overall true positive percentage of 83.8%, a true negative percentage of 72.0%, and a correct move percentage of 70.6%.

#### Subjects A and F: 2-D control with a single electrode and no referencing

To test if good single-channel control could be retained without Laplacian derivation referencing, we performed an offline analysis of the data from Subject A, using the recording from the threshold-setting task to set a threshold and the recording from the cursor control task to determine classification accuracy. As before, the electrode and frequency band were C3 and 20–24 Hz. The only change was eliminating the Laplacian derivation referencing, and using the raw signal from C3.

From this analysis, we calculated the following results. The total number of yes/no answers was 104, consisting of 34 true positives, 27 false positives, 36 true negatives, and 7 false negatives. This corresponds to a true positive percentage of 82.9% and a true negative percentage of 57.1% (chance = 50% for each). The overall correct cursor move percentage was 49.1% (chance = 31.2%).

**Figure 6:**
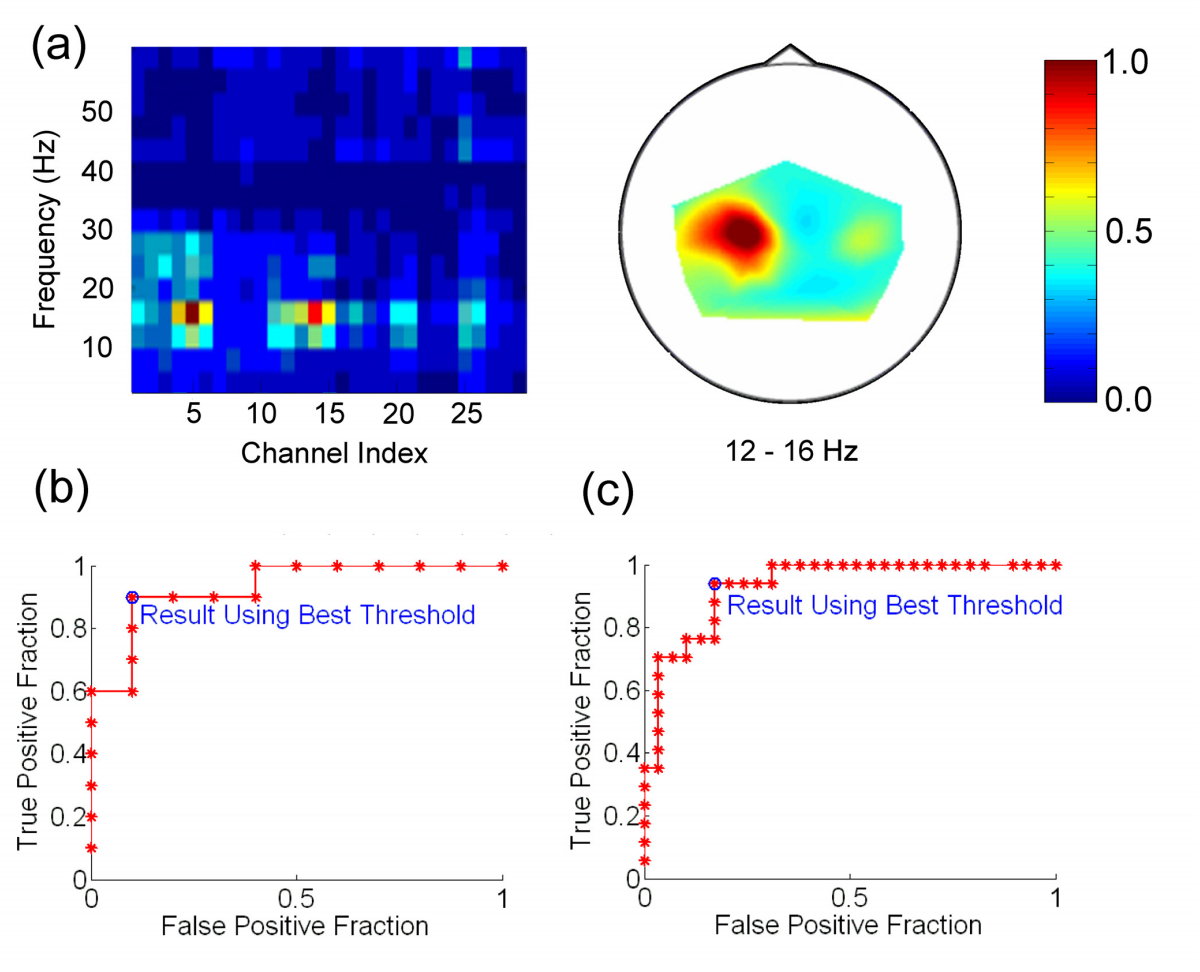
**Subject F using real movement with no Laplacian derivation referencing (see text)**. (a) Bhattacharyya plots. Left: Channel-frequency plot. Right: Head topography plot for the 12–16 Hz bin. (b) ROC curve from threshold-setting task. (c) Refined ROC curve after performance of the cursor control task with the adaptive threshold enabled.

We also performed one online test of our paradigm with true single-channel control. For Subject F, a healthy subject using real movement, we did not use Laplacian derivation referencing. Figure 6(a) shows Bhattacharyya distance plots for Subject F. We selected the 12–16 Hz frequency bin and C3 electrode for control. Using this channel/bin, the subject performed the threshold-setting and cursor control tasks. Rather than beginning a hand movement for each response and ceasing the movement to answer "no," the subject chose to perform hand movement to answer "yes," and to abstain from such movement to answer "no." The ROC curve from the threshold-setting task, using 20 prompts rather than 30, is shown in Figure 6(b). This curve showed good quality classification, with a low value of the distance defined in (2). After the threshold-setting task, the subject proceeded to play the cursor control game with an adaptive threshold feature enabled. The adaptive threshold feature allowed the program to learn as the subject played the cursor control game, by recalculating the ROC curve and optimum threshold after every yes/no answer for which there was a unique good choice. The resultant ROC curve is shown in Figure 6(c). Finally, the subject performed the cursor control task with the adaptive threshold disabled. The subject demonstrated good cursor control, with a true positive percentage of 83.3%, a true negative percentage of 89.8%, and a correct move percentage of 77.4%.

## Discussion

### Binary 2-D cursor control with hand movement

Overall, control was good with healthy subjects using real hand movement. Accuracy for Subject B might have been even higher had the optimum channel/bin been customized for this subject, whose Bhattacharrya plots indicated better classification at slightly higher frequencies around 28–32 Hz, and whose control was less accurate than that of the other subjects. The very good results from Subject D, for whom the optimum channel/bin was customized, provide further support for the benefit of such individualized calibration. However, the downside of such calibration is that it requires all 29 EEG channels to initially be attached to the subject, even though most of the channels ultimately may not be used for control.

The high control accuracy seen with all subjects demonstrates that the binary method with hand movement is effective and robust. The method requires remarkably little user training. Each subject practiced with the threshold-setting task prior to performing the cursor control task, and Subject C also practiced briefly with the cursor control task before achieving the results given in Table 1. However, all four naïve subjects achieved the control accuracies reported in Table 1 within the first 2 hours of their experience with the paradigm.

These results are consistent with subsequent experiments using healthy subjects with real movement [21].

### Supplementary tests

#### Subject B: 2-D control with motor imagery (no hand movement)

If the system is to be useful to individuals who are so severely disabled that they entirely lack the ability to make voluntary movements (locked-in syndrome), it must work in the absence of hand movements. The state of the art in BCI research is to use motor imagery in place of real movement to attempt to replicate the effects of paralysis. This eliminates the sensory feedback component of the EEG signal, but also may not provide a realistic motor EEG signal (i.e., individuals with paralysis attempt to move but cannot, whereas individuals imagining movement are actively refraining from moving, so while both groups of subjects might be expected to exhibit associated premotor activity, the associated primary motor activity in the subjects using motor imagery would probably not be as robust as in the paralyzed subjects).

The estimated cursor movement accuracy of 53.3% for Subject B using motor imagery obviously represents less accurate control than for real movement, but some control remained. The low degree of control with motor imagery was reflected by the subject's frustration. These results suggest that the paradigm in its current form would have limited usefulness for individuals with locked-in syndrome. However, subsequent testing has shown that the utility of our method under conditions of motor imagery should not be discounted, especially when control is measured by the subject's ultimate ability to attain the target, rather than by the accuracy of each cursor movement step [21].

#### Subject E: 2-D control with weak movement

We hoped that testing the paradigm with a PLS patient would provide an indication as to whether our system might benefit individuals who have motor disabilities but retain limited motion. We also expected that testing with a patient who had limited motion would reduce any artifactual sensory component of the controlling EEG signal, further supporting our assertion that the controlling signal during hand movement results from motor activity rather than sensory feedback.

Based on the results of testing with Subject E, we concluded that she had good control despite her PLS. This suggests that our system might be useful to individuals who have movement disabilities but are not locked-in. This conclusion is consistent with the results of subsequent testing on individuals with motor disability secondary to amyotrophic lateral sclerosis (ALS) or hemorrhagic stroke [21]. Further investigations on a larger patient population are required; in particular, the paradigm should be tested with subjects who are severely affected.

#### Subjects A and F: 2-D control with a single electrode and no referencing

While Laplacian-referenced single-channel control requires only five EEG electrodes to be attached to the subject, "true" single channel control, using only one electrode, would require even less hardware setup. However, with true single-channel control, no Laplacian derivation referencing can be used. We expected that eliminating such referencing would significantly degrade control, which is why we used the referencing in our primary procedure.

Clearly, Subject A's overall correct cursor move percentage of 49.1% without Laplacian referencing (offline, post-hoc) represents a large degradation of control compared to when Laplacian derivation referencing was used for the same session (online, real-time). However, some control above chance level was retained.

It is also reasonable to expect that results would be better in an online control scenario, since the subject would have the opportunity to dynamically adjust strategy. This may have been one component of what happened in the online test with Subject F, who demonstrated good cursor control in a real-time scenario, despite the absence of Laplacian referencing. These results support the idea that, under appropriate conditions, achieving decent true single-channel control is practical. However, consistent testing across multiple subjects is needed. Ultimately, the specific application will dictate whether the reduction in hardware complexity justifies the compromise in functionality associated with true single-channel control.

### Comparative accuracy, speed, and ease of control

Like the method of Piccione et al. [15], our system applies a known approach to obtaining information from EEG (in our case, band power measurement; in Piccione's, evoked potential detection) in a novel paradigm to address the challenge of two-dimensional cursor control. Unlike Piccione's 2-D control method, our 2-D control method classifies each individual user response into one of two possible classes (yes or no), with two user responses per cursor step. Piccione's method classifies each user response into one of four classes (up, down, left, or right), with one user response per cursor step. At chance level, our method would equal Piccione's in accuracy for a typical cursor move. Chance level for a correct cursor movement under Piccione's method is equal to chance level for the correct classification of an individual user response, or 1/4. Chance level for a typical correct cursor movement under our method is equal to the product of the chance levels for each of the two responses being classified correctly, or (1/2)^2 ^= 1/4. We originally expected the band power signal upon which response classification is based in our paradigm to be more robust than the P-300 signal upon which response classification is based in Piccione's paradigm. Therefore, we originally hypothesized that our paradigm would yield more accurate control than Piccione's paradigm, while still providing equivalent stepwise 2-D cursor control at a roughly equivalent speed.

From our results, the average accuracy of our system was 86.1%, with one cursor move occurring approximately once every 15 s (≈ 8 bits/min). Comparatively, Piccione's system had an average accuracy of 76.2% and a bit rate of 7.59 bits/min with healthy trained subjects [15]. Based on these results, our method appears to have a higher accuracy at a slightly higher speed than Piccione's method, while providing 2-D cursor control of an equivalent nature (occurring in sequential steps as guided by user selection of a dimension and direction at each step).

The bit rate of our system, while at least comparable to Piccione's and other accepted BCIs, might still be improved through subject training to build response proficiency, allowing for shorter pauses between stages of the paradigm. Alternatively, if both bits needed for a cursor move could be collected simultaneously (e.g., by two channels – one over the sensorimotor cortex of each hemisphere), bit rate might be increased. Geng et al. describe one promising "parallel" 4-class BCI that uses two binary classifiers obtained simultaneously during hand and foot movement, although the computational method used is more complex than our threshold-based classification [16]. Further evaluation is needed to assess whether the bit rate of our system can be improved without sacrificing accuracy.

When considering the speed and efficiency of our method, it should be noted how our cursor control method is conceptually distinct from that employed in EEG-based decision trees. In such trees, real-time analysis of band power controls one dimension of cursor movement while cursor movement in the other dimension proceeds at a constant rate. This continues until the cursor reaches the edge of the screen, at which point one of multiple targets is achieved, and a single selection is thereby made. If there are two available targets (the scenario, closest to that of our paradigm, in which accuracy is expected to be highest), this entire process constitutes a single binary selection, ultimately yielding only one bit of information. In our paradigm, two complete binary selections are used to determine both the dimension and direction of each cursor movement, and only then does the cursor move one space in the determined dimension/direction. Thus, the information obtained by our paradigm for each movement of the cursor by one space is twice the amount of information that would be obtained from conducting an entire two-choice decision tree cursor control paradigm. However, our paradigm may take significantly less time per cursor move than even a single typical decision tree selection. This should be considered when judging the speed of our method.

Also noteworthy is that our method provides both the higher accuracy associated with binary selections and a straightforward means of correcting cursor movement errors: if the cursor is moved in an undesired direction, the user may move it back to the previous position on the subsequent move, or may continue movement toward the desired target using an alternate path. Contrast this with error correction in a decision tree selection, which requires a separate "undo" option in addition to the at least two other options from which the user is expected to select. Because of this "undo" option, decision trees that allow error correction must classify each selection into one of at least three possible classes, resulting in lower selection accuracy than could be achieved from a binary classification.

Comparison of our system's accuracy and speed with contemporary 2-D EEG BCI systems other than Piccione's is less straightforward, due to necessarily diverse methods of evaluating accuracy and speed. The two-band system of Wolpaw et al. [13] allows subjects to take up to 10 s to attain one target of eight. Because the two dimensions of movement are controlled simultaneously, success was measured not by the accuracy of each cursor step, but rather by whether the subject could attain the target by any path within the 10 s time limit. The average success rate of four subjects was 82%, with an average successful target acquisition time of 2.8 s. The first target attained by each subject was strongly correlated with the intended target (p < 0.001). In the system of Trejo et al. [7], which uses SSVEP control, the direction of cursor motion is recalculated every 250 ms, with control lags of 1–5 s. Because during each 250 ms movement period, movement is in only one of the four directions (left, right, up, or down), Trejo's 2-D control method is similar to both our method and that of Piccione in that it does not provide simultaneous control of the two dimensions of movement. However, because each movement period is only 250 ms, the method gives the illusion of simultaneous control of the two dimensions. Trejo's method yielded an average movement accuracy across three subjects of 76%.

While the binary control method of our system is less naturalistic than is ideal, all EEG-based control systems suffer from being somewhat awkward to use, and many extant EEG BCI systems are, like ours, externally paced. Our synchronous approach may be less naturalistic than is self-paced control, but the approach allows classification accuracy to be high despite our simple computational method. (Consider that determining when a subject is answering is a significant challenge in self-paced systems; the best asynchronous EEG switch developed by the Neil Square Society as of 2005 had a 73% mean activation rate and a 2% mean false positive error rate across four subjects [22].) Scherer et al. described a self-paced EEG BCI for 2-D navigation in a virtual environment [23]. While their system offers the potential of naturalistic pacing, it still features somewhat unnatural control methods (hand, foot, and tongue motor imagery), as well as variable accuracy and high computational demand. Thus, self-pacing is not the only obstacle to naturalistic control. We believe that our system is not unacceptably less natural to use when measured against the state of the art in EEG BCI.

We also believe that our system may be less fatiguing than are some other EEG BCI systems, particularly those that rely on visually evoked potentials. However, further testing is required to determine this conclusively.

## Conclusion

We have demonstrated a method of achieving simple and accurate real-time 2-D cursor control from a single channel of EEG with Laplacian referencing obtained from four additional channels during naive subject hand movement.

A primary asset of our system is its simplicity. Computation is limited to straightforward power calculations over externally paced time windows. With only one Laplacian-referenced channel used for control, only five electrodes need to be attached to the subject. Overall, the system needs less complex hardware and less computational capability than do many EEG BCI systems.

Our system also offers the significant benefit of requiring very little user training for effective control. Each of our subjects achieved his or her reported level of control in the first day of using the paradigm. Essentially, the paradigm allowed naïve subjects to have good control of the cursor immediately. The computer program also did not require a lengthy training period; it needed less than five minutes to complete the initial threshold-setting task. The user training requirements of the paradigm are among the lowest of EEG control systems; at the other extreme, some systems require months of user training before effective control is attained [4-7,13,14]. Blankertz et al. described an EEG BCI system that, like ours, has the benefit of needing very little user training [24]. However, this system does not have the advantage of our system's computational and hardware simplicity.

The results of our supplementary experiments further support the utility of the paradigm. Results from the subject with PLS show that control can be achieved under this condition, suggesting that the system might be useful to individuals with impaired movement. This is consistent with subsequent findings from another study [21].

The supplementary data shows that some control is possible using motor imagery. However, this control was relatively poor. In general, the performance of motor imagery is highly related to how well subjects can imagine movements vividly. As motor imagery is not the main aim of the current study, we did not train subjects to optimize motor imagery, but such training might improve future results. Subsequent study has suggested more promise for our paradigm using motor imagery [21].

We believe that a BCI that uses physical movements (if available) is potentially beneficial to individuals with neurological conditions other than locked-in syndrome. The locked-in condition may only present in the late stages of patients with ALS. However, ALS progresses quickly and usually the patients die within 3–5 years after diagnosis [25]. Most of the time, ALS patients experience symptoms of stiffness and may be unable to make reliable muscle contractions although they are still able to move [26]. We believe combining BCI with limited motor function may be suitable for these patients. Such a combined approach is not unprecedented; for example, SSVEP paradigms like Trejo's require that subjects have the voluntary eye movement control necessary to selectively attend to stimuli [7].

Supplementary results from the two subjects without Laplacian referencing show that diminished control can be achieved under this condition, suggesting that control might be practical with only a single EEG electrode attached. Because of the associated loss of accuracy, this should probably only be done if such added simplicity is a compelling consideration.

Two-dimensional cursor control from binary classification of EEG signals is simple, accurate, and requires remarkably little training. Because of its computational and hardware simplicity, the technique could potentially be implemented relatively easily in an in-home setting. For immediate purposes, an easy-to-use in-home cursor control game might be beneficial to individuals who need to practice controlling their EEG rhythms but who would rather not make repeated trips to an EEG laboratory. With further development, binary cursor control, alone or combined with other technologies, could potentially have practical application to device control and communication.

## Abbreviations

EEG: electroencephalography; BCI: brain-computer interface; 2-D: two-dimensional; PLS: primary lateral sclerosis; ECoG: electrocorticography; MEG: magnetoencephalography; fMRI: functional magnetic resonance imaging; ERD: event-related desynchronization; 1-D: one-dimensional; SSVEP: steady-state visual evoked potential; EMG: electromyography; FFT: fast Fourier transform; ROC: receiver operating characteristics; ALS: amyotrophic lateral sclerosis.

## Competing interests

The authors declare that they have no competing interests.

## Authors' contributions

TAK conceived, designed, and developed the binary 2-D cursor control paradigm. TAK originated the idea of the paradigm, including serial dichotomous response classification using a threshold set based on a single-channel-frequency feature, as well as the application of this classification to 2-D cursor control. TAK defined and refined the paradigm's features, and wrote the Matlab code to comprehensively implement the paradigm within OB's software system for EEG data acquisition and processing. TAK also assisted with data collection, performed the data analysis, and drafted and revised the manuscript. OB developed the Matlab-based software system for EEG data acquisition and processing within which TK's paradigm-specific Matlab program was implemented. OB also collected data, refined the specific application of the paradigm to the study participants, assisted with data analysis, and assisted with critically revising the manuscript. CSH provided invaluable guidance and critical input for revising the manuscript. PL assisted with data collection and analysis, and assisted with critically revising the manuscript. SJF recruited study participants, collected data, and assisted with refining the specific application of the paradigm to the study participants. SV contributed substantially to the hardware setup and data collection for all study participants. MH is the Chief of the Human Motor Control section of NINDS and assisted with critically revising the manuscript. All authors read and approved the final manuscript.

## Supplementary Material

Additional file 1 **Example Video**. Demonstrates the general sequence of a typical cursor control game. No audio. Click here for file

Additional file 2 **Overview**. Summarizes the binary control computational method. Click here for file
