# Supplementary material for: A binary method for simple and accurate two-dimensional cursor control from EEG with minimal subject training
Source: J Neuroeng Rehabil. 2009 May 6;6:14. doi: 10.1186/1743-0003-6-14 (PMC2683855; doi:10.1186/1743-0003-6-14)
Supplement: Additional file 2 — Overview. Summarizes the binary control computational method. [file 1743-0003-6-14-S2.pdf]

The binary control computational method detailed

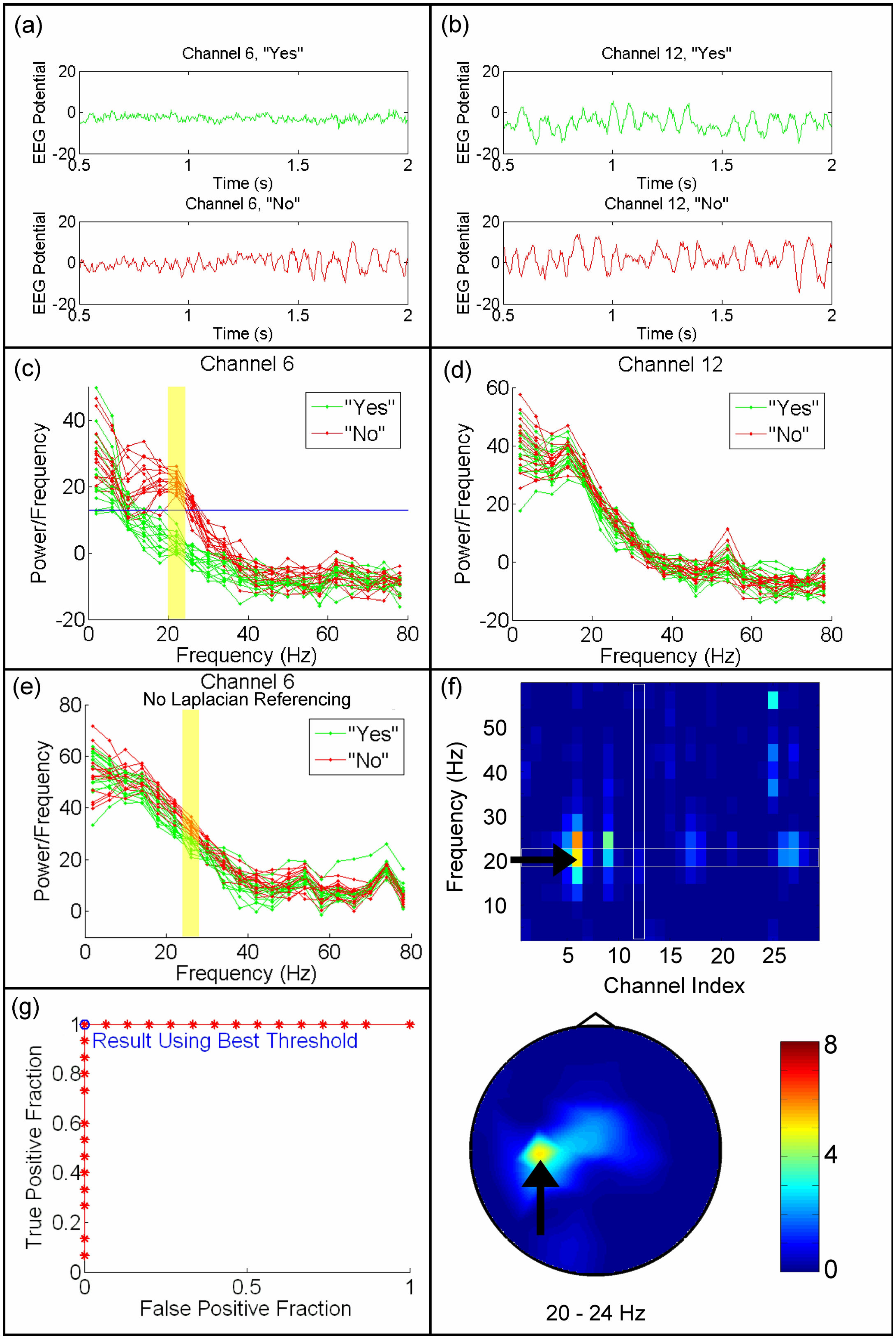

EEG signals are recorded during subject responses. To respond “yes,” a subject makes a continuous right hand movement while the prompt is green. To respond “no,” the subject refrains from such movement. The EEG is recorded starting at 0.5 s after the prompt turns green (to allow for response time), until 2 s after the prompt turns green.

Single examples of Laplacian derivation referenced EEG traces recorded from Subject C are shown in (a) and (b), where time 0 corresponds to the onset of a green prompt. Traces from channel 6 (the C3 electrode, used in this investigation) are shown in (a), and traces from channel 12 (an electrode distal to C3, chosen arbitrarily for this example) in (b). Top traces in (a) and (b) correspond to intended “yes” responses, and bottom traces to intended “no” responses. The two classes of responses are more visually distinct on channel 6 (a) than channel 12 (b). This difference is also reflected by the spectra of the signals.

Shown in (c) and (d) are Welch estimates of the power spectral densities of the 15 intended “yes” and 15 intended “no” responses from Subject C’s training session. Intended “yes” and “no” signals from channel 12 (d) have similar spectral densities, whereas the “yes” signals from channel 6 have spectral densities that are distinctly lower than those of “no” signals in the 20-24 Hz frequency band highlighted in (c). The lower powers of “yes” signals in this channel/band are attributable to ERD. As an aside, (e) shows the spectral density on channel 6 if Laplacian derivation referencing is not used. Without such referencing, the “yes” and “no” signals are best distinguished in the highlighted 24-28 Hz band, but this distinction is of a lower quality than in (c), where Laplacian derivation referencing was used.

From (c), the fact that the two response classes are highly distinguishable in the 20-24 Hz band of channel 6 is reflected quantitatively by the high value of the Bhattacharyya distance for that channel/band, indicated by the black arrow on the top plot of (f). Conversely, for channel 12 in this plot, outlined by the vertical white box, all Bhattacharyya distances are low, reflecting the fact, illustrated in (d), that the yes and no classes are not clearly distinguishable based on the spectral densities of the corresponding EEG recordings made on channel 12. The bottom plot of (f) shows a head topography plot of the Bhattacharyya distances for only the 20-24 Hz band, corresponding to the row on the top plot outlined by the horizontal white box. The position of channel 6 on the bottom plot is indicated by the black arrow.

To use the 20-24 Hz band of channel 6 for control, the threshold-setting task is performed for this channel/band. The program constructs an ROC curve for this channel/band to set an optimum threshold above which responses are classified as “no” and below which responses are classified as “yes.” This optimum threshold corresponds to the blue line in (c). As can be seen in (c), applying this threshold in the highlighted band allows classification of the threshold-setting data to be perfect in this example. The perfect classification using the optimum threshold appears as the point indicated by the blue circle on the ROC curve generated in the threshold-setting task (g). Note that no such “perfect” threshold is possible to define for the non-referenced signals in (e).

After the optimum threshold is determined from the threshold-setting task, it is used for the cursor control paradigm. For each response, Laplacian referenced EEG is recorded on channel 6 and its power spectral density in the 20-24 Hz band is compared to the previously determined optimum threshold to classify the response. If the power value falls above threshold, the response is classified as a “no,” and if the power value falls below threshold, the response is classified as a “yes.”
